# Traumatic endophthalmitis caused by *Nocardia kruczakiae* in a patient with traumatic eye injury

**DOI:** 10.1186/s12348-015-0067-7

**Published:** 2015-11-25

**Authors:** Rafael Barraquer Compte, Hernán Martínez-Osorio, Gema Carrasco, Betty Lorente, Javier Elizalde, Sylvia Valdezate, Ramón Lorente, Emilio Iglesias, Juan Antonio Saez-Nieto

**Affiliations:** Centro de Oftalmología Barraquer, Muntaner 314, 08021 Barcelona, Spain; Institut universitari Barraquer, Barcelona, Spain; Servicio de Bacteriología, Centro Nacional de Microbiología, Instituto de Salud Carlos III, Majadahonda, Madrid, Spain; Servicio de Oftalmología, Complexo Hospitalario Universitario, Ourense, Spain

**Keywords:** *Nocardia kruczakiae*, Bacterial endophthalmitis, Multi-targeted identification

## Abstract

**Background:**

We describe a case of traumatic ocular endophthalmitis caused by *Nocardia kruczakiae* after vegetable trauma in an immunocompetent child.

**Findings:**

A 5-year-old boy suffered from a trauma with a palm tree leaflet. Two months later, he was diagnosed with traumatic infectious uveitis and intumescent cataract with anterior capsule rupture. Intensive treatment with systemic and topical vancomycin, ceftazidime and methylprednisolone began. After 1 month, he underwent phacoemulsification with intraocular lens implantation (IOL).

After some episodes of reactivation, he was diagnosed with traumatic nocardial endophthalmitis from aqueous humour samples. Several operations and specific antibiotic therapy resolved the infection.

**Conclusions:**

In cases of traumatic endophthalmitis and several recurrences, it is extremely useful to make an etiologic diagnosis in order to treat the patient with specific antibiotics.

## Findings

A 5-year-old boy suffered trauma in his left eye (OS) by a palm tree leaflet in October 2009. Two months later, he was diagnosed with traumatic infectious uveitis and intumescent cataract with anterior capsule rupture. Treatment with systemic and topical vancomycin, ceftazidime and methylprednisolone began. In January 2010, the patient underwent phacoemulsification with intraocular lens implantation (IOL). Aqueous humour samples were cultured with negative results. Every reduction in treatment led to several episodes of anterior uveitis. Inflammation continued although systemic and topical clarithromycin plus antimycotic treatment and antiglaucoma eye drops were prescribed.

**Fig. 1:**
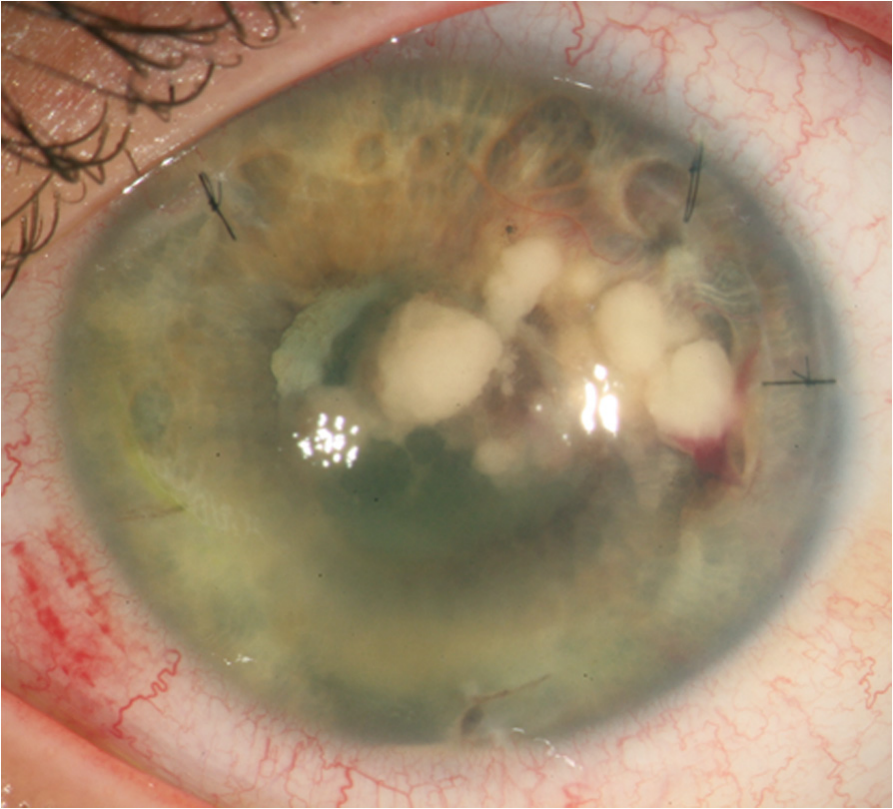
Left eye N. krckzakiae endophthalmitis. The slit lamp examination shows yellowish nodules in anterior chamber above iris and intraocular lens

The patient was admitted to our centre (*Centre Oftalmología Barraquer*) in July 2010. He was being treated with topical dexamethasone, atropine, ciprofloxacin, voriconazole and antiglaucoma eye drops: oral acetazolamide, deflazacort, fluconazole, omeprazole and calcium carbonate/colecalciferol. Visual acuity (VA) was 0.95 in the right eye and 0.1 in the OS. Examination revealed yellowish nodules above the iris and IOL (Fig. 1). Ultrasounds only revealed a slight inflammatory reaction. The patient’s medical history was unremarkable. Anterior vitrectomy and IOL and capsular bag removal were performed, adding intraocular vancomycin and ceftazidime injection. The latter treatment was continued and oral clarithromycin restarted. Now, the aqueous humour culture showed an aerobic gram-positive bacillus compatible with Actinomycetes. Antimycotic treatment was stopped, and topical polymyxin B plus trimethoprim and oral trimethoprim/sulphamethoxazole were therefore provided. Two weeks after vitrectomy, the patient showed reduction of inflammation, but retinal detachment with macular involvement was diagnosed. Scleral buckling, endophoto-coagulation and pneumatic retinopexy were performed.

The Actinomycetes was further identified and antimicrobial susceptibility tested [1]. The 16S RNA, 65-kDa heat-shock protein (*hsp65*), β-subunit type II DNA topoisomerase (*gyrB*) and RNA polymerase subunit β (*rpoB*) genes were examined [2–4]. Sequences were compared with those in the GenBank (http://www.ncbi.nlm.nih.gov/BLAST) and Bacteria Identification Bioinformatics (BIBI) databases (http://umr5558-sud-str1.univ-lyon1.fr/lebibi/lebibi.cgi). Similarities of ≥99.0 % were deemed to denote the same species.

In terms of 16S, the bacterium was most similar (99.0 %) to *Nocardia nova* IFM 0272. Similarity with *N. nova* DSM 44481 and DSM 43207 [5] was lower at 98.7 %. Ninety-eight percent similarity was detected with the sequences for *Nocardia africana*, *Nocardia aobensis*, *Nocardia cerradoensis*, *Nocardia kruczakiae* and *Nocardia veterana*. The *hsp65* gene showed 99 % similarity with respect to those of *N. aobensis* DSM 44805, *N. nova* DSM 44481, *N. veterana* NRRL B-24136 and *N. kruczakiae* DSM 44877 [5].

The *gyrB* gene was most similar (99 %) to that of *N. kruczakiae* W9710/DSM 44877, *N. aobensis* DSM 44805 and *N. cerradoensis* W8368 [3]. Among four detected polymorphisms (*N. kruczakiae* DSM 44877 numbering), one produces the replacement Trp(TGG) → Cys(TGC). A fully matching *rpoB* sequence was obtained with *N. kruczakiae* DSM 44877. Similarity fell to 99 % with respect to *N. aobensis* DSM 44805 and *N. cerradoensis* DSM 44546 and to 98 % with respect to *N. nova* OAHPP13857-1633 [5]. The studied bacterium, CNM997/10, was therefore identified as *N. kruczakiae*. The sequences were deposited in GenBank under accession numbers JX443642–JX443645.

Ocular inflammation resolved after 2 months with specific treatment. Two months after retinal surgery, the patient underwent iris reconstruction, secondary IOL fixation and Ahmed valve implantation. VA improved to 0.2. Three years after treatment discontinuation, no inflammation was observed.

### Discussion

Ocular infections caused by *Nocardia* species previously unknown in immunocompetent patients have recently been reported [6, 7]. The cornea and anterior chamber of the eye are immune-privileged tissues; this may explain the appearance of ocular infections or surgically related endophthalmitis in such patients [6, 8].

This report describes an immunocompetent child who suffered ocular trauma by a palm tree leaflet inoculating *N. kruczakiae*. Nocardial infection should be considered in patients with such plant-inflicted trauma since 31–67 % of postoperative nocardial endophthalmitis have occurred in those living in rural areas [6, 9]. Initial management with rounds of corticosteroids probably encouraged chronic infection. Long corticosteroid treatment predisposes patients to nocardial endophthalmitis [9]. A large proportion of patients (75–83 %) show nodules on the corneal endothelium or on the iris; however, the posterior segment is usually normal or only slightly involved [6, 9]. Surgical procedures are frequently used to eradicate nocardial endophthalmitis. The outcome of nocardial endophthalmitis can be poor due to its delayed presentation and extensive involvement of the anterior chamber. The present patient showed an improvement to a VA of 0.2 OS from 0.1, despite late diagnosis and the retinal detachment that occurred after IOL removal.

*N. kruczakiae* is difficult to distinguish from *N. africana*, *N. nova* and *N. veterana* by phenotyping [2] but can be identified by molecular technique even when the samples available are very small and patients have undergone treatment with antibiotics [7, 10]. 16S analysis commonly provides a definitive identification, but certain closely related species cannot be differentiated, a consequence of the low level of interspecies polymorphism and the existence of multiple and different copies of 16S in *N. nova* [11, 2]. Indeed, several species, such as *N. africana*, *N. aobensis*, *N. cerradoensis*, *N. nova*, *N. kruczakiae* and *N. veterana*, cluster together even when examined by multilocus sequence typing [5].

The *rpoB* and *gyrB* genes are known to show greater diversity than 16S and *hsp65* and therefore allow for more precise identification [5]. Those of the causal agent were found similar (100 and 99.3 %, respectively) to those of the *N. kruczakiae* DSM 44877, confirming that it belonged to this specie.

*N. kruczakiae* CNM997/10 also showed the same susceptibility profile to that first described for *N. kruczakiae* ATCC BAA-280 [2], except for ampicillin (Table 1). Treatment with amikacin, clarithromycin, imipenem, linezolid and trimethoprim/sulphamethoxazole would therefore appear appropriate.

**Table 1:** Antimicrobial susceptibility of *N. kruczakiae* CNM997/10, the causal agent of endophthalmitis in the present patient

Antimicrobial agent	MIC (μg/ml)^a^	Susceptibility^b^	Resistance breakpoint^c^
Primary^c^			
Amikacin	0.19	S	≥16
Amoxicillin/clavulanic acid	≥256	R	≥32/16
Ciprofloxacin	≥32	R	≥4
Clarithromycin	0.5	S	≥8
Imipenem	0.5	S	≥16
Linezolid	1	S	≤8
Trimethoprim/sulphamethoxazole	2/38	S	≥4/76
Tobramycin	≥256	R	≥16
Others^c,d^			
Ampicillin	≥256	R	≥32
Cefotaxime	≥32	R	≥64
Meropenen	8	–	Na
Gentamicin	8	I	≥16
Tetracycline	≥256	–	Na
Chloramphenicol	≥256	–	Na
Clindamycin	0.03	S	≥4
Quinupristin/dalfopristin	≥32	–	Na
Vancomycin	≥32	–	Na
Teicoplanin	≥256	–	Na

^a^Minimum inhibitory concentration; ^b^S and R, susceptible and resistant; ^c^Resistance and susceptibility breakpoints are those recommended by the CLSI 2011 M24-A2, and ^d^by the NCCLS 2003 M24-A; Na, not available

The suggested empirical treatment for severe ocular bacterial infections is topical and intravitreal vancomycin and ceftazidime [12]. Unfortunately, this led to the recurrence of iris nodules in this patient. His endophthalmitis was finally brought under control after surgical removal of the lens-bag complex with associated inflammatory materials, which allowed the detection of *N. kruczakiae*. Specific treatment for 8 weeks resolved the condition.

*N. kruczakiae*, previously described as a causal agent of pneumonia [2], is reported here as the causal agent of ocular endophthalmitis. Ophthalmologists should be aware of infections caused by *Nocardia* and suspect nocardial endophthalmitis after plant-inflicted trauma.
